# Intravoxel incoherent motion diffusion-weighted imaging for predicting kidney allograft function decline: comparison with clinical parameters

**DOI:** 10.1186/s13244-024-01613-y

**Published:** 2024-02-16

**Authors:** Wei Wang, Yuanmeng Yu, Jinsong Chen, Longjiang Zhang, Xue Li

**Affiliations:** 1 National Clinical Research Center of Kidney Diseases, Jinling Clinical Medical College of Nanjing Medical University, Nanjing, 210002 Jiangsu China; 2 Department of Nephrology, Shanghai Tenth People’s Hospital, Shanghai, China; 3 Department of MRI, The First People’s Hospital of Yunnan Province, The Affiliated Hospital of Kunming University of Science and Technology, Kunming, 650032 Yunnan China; 4 Department of Medical Imaging, Jinling Hospital, Clinical School of Southern Medical University, Nanjing, 210002 Jiangsu China; 5 National Clinical Research Center of Kidney Diseases, Jinling Hospital, Nanjing University School of Medicine, Nanjing, 210002 Jiangsu China; 6 Department of Diagnostic Radiology, Jinling Hospital, Nanjing University School of Medicine, Nanjing, 210002 Jiangsu China

**Keywords:** Diffusion magnetic resonance imaging, Glomerular filtration rate, Kidney transplantation, Proteinuria

## Abstract

**Objective:**

To evaluate the added benefit of diffusion-weighted imaging (DWI) over clinical parameters in predicting kidney allograft function decline.

**Methods:**

Data from 97 patients with DWI of the kidney allograft were retrospectively analyzed. The DWI signals were analyzed with both the mono-exponential and bi-exponential models, yielding total apparent diffusion coefficient (ADC_T_), true diffusion (D), pseudo-diffusion (D*), and perfusion fraction (fp). Three predictive models were constructed: Model 1 with clinical parameters, Model 2 with DWI parameters, and Model 3 with both clinical and DWI parameters. The predictive capability of each model was compared by calculating the area under the receiver-operating characteristic curve (AUROC).

**Results:**

Forty-five patients experienced kidney allograft function decline during a median follow-up of 98 months. The AUROC for Model 1 gradually decreased with follow-up time > 40 months, whereas Model 2 and Model 3 maintained relatively stable AUROCs. The AUROCs of Model 1 and Model 2 were not statistically significant. Multivariable analysis showed that the Model 3 included cortical D (HR = 3.93, *p* = 0.001) and cortical fp (HR = 2.85, *p* = 0.006), in addition to baseline estimated glomerular filtration rate (eGFR) and proteinuria. The AUROCs for Model 3 were significantly higher than those for Model 1 at 60-month (0.91 vs 0.86, *p* = 0.02) and 84-month (0.90 vs 0.83, *p* = 0.007) follow-up.

**Conclusions:**

DWI parameters were comparable to clinical parameters in predicting kidney allograft function decline. Integrating cortical D and fp into the clinical model with baseline eGFR and proteinuria may add prognostic value for long-term allograft function decline.

**Critical relevance statement:**

Our findings suggested that cortical D and fp derived from IVIM-DWI increased the performance to predict long-term kidney allograft function decline. This preliminary study provided basis for the utility of multi-b DWI for managing patients with a kidney transplant.

**Key points:**

• Both clinical and multi-b DWI parameters could predict kidney allograft function decline.

• The ability to predict kidney allograft function decline was similar between DWI and clinical parameters.

• Cortical D and fp derived from IVIM-DWI increased the performance to predict long-term kidney allograft function decline.

**Graphical Abstract:**

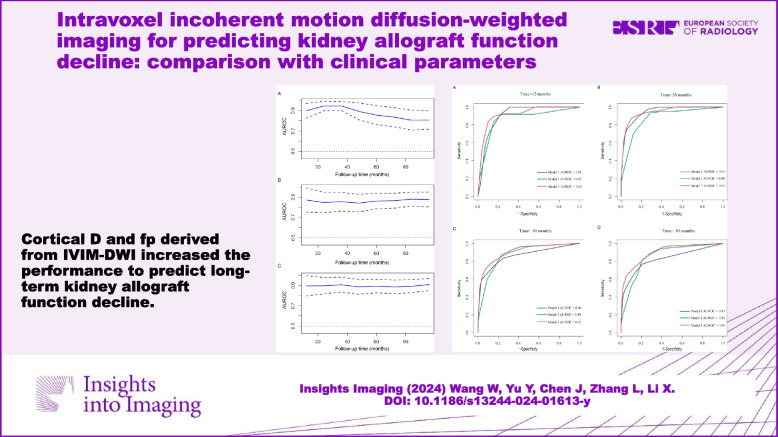

## Introduction

Kidney transplantation is an effective surgical procedure for managing patients with end-stage kidney diseases, which has been shown to confer superior quality of life and clinically relevant outcomes compared to dialysis [1]. Proper postoperative management and accurate prognostication have emerged as essential elements for maintaining the longevity of kidney allografts. From this perspective, biomarkers that could inform risk of kidney function decline would facilitate clinical judgment and ascertain precise prognostication. Currently, serum creatinine and proteinuria are the main clinical measures used to assess renal function.

Diffusion-weighted imaging (DWI) is a robust imaging technique probing the displacement of water molecules that are reflective of underlying microstructural changes. Accumulating evidence suggests that DWI of the native and transplanted kidneys is technically feasible and reproducible [2]. Recent advances in intravoxel incoherent motion DWI (IVIM-DWI) have enabled the separation of pseudo diffusion from true diffusion by using the biexponential model for signal analysis. Prior cross-sectional studies have consistently shown that DWI parameters may assist with characterizing pathologic changes, especially interstitial fibrosis, in both the native and the allograft kidneys [3, 4].

To the best of our knowledge, the majority of available DWI studies on kidney allografts have been cross-sectional in design, and it remains unknown whether DWI parameters are predictive of kidney allograft survival. If so, what is the additive prognostic value of the DWI parameters, in addition to clinical biomarkers? To bridge this gap, this study aimed to evaluate the predictive value of DWI parameters for kidney allograft function decline and to clarify the additive long-term prognostic value of these DWI parameters.

## Materials and methods

### Study population

This single-center retrospective study was performed after obtaining approval from the local ethics committee. Informed consents from patients were waived due to the retrospective and non-interventional nature of this analysis. Data from a total of 115 adult patients evaluated for clinically driven indications, including post-transplant rising serum creatinine, proteinuria, or the appearance of donor-specific antibodies suspicious for allograft rejection, were retrieved and scrutinized. These patients underwent posttransplant multi-b DWI from March 2014 to October 2015 as part of a comprehensive evaluation that also comprised laboratory measurements. A total of 18 cases were excluded for suboptimal imaging quality (*n* = 8), incomplete clinical or laboratory data (*n* = 7), and no follow-up (*n* = 3), leaving a final analysis of available data from 97 patients.

### DWI acquisition and image analysis

MRI was performed with a 3.0 Tesla clinical imager (General Electric, Milwaukee, WI, MR750, USA) equipped with a 32-channel body coil after the patient had been fasting for at least 4–6 h. Coronal T1-weighted and axial T2-weighted images were routinely acquired for anatomic depiction. Axial DWI images were acquired with a single-shot echo-planar imaging sequence using the respiration-triggered technique. A total of 10 nonzero *b*-values (10, 30, 50, 70, 100, 150, 200, 400, 800, and 1000 s/mm^2^) were applied with the following parameters: repetition time 2857 msec, echo time 87.2 msec, field of view 38 × 30.4 cm, matrix 256 × 128, slice thickness 6 mm, number of slices 15, and number of excitations 2. Diffusion-sensitizing gradients were applied along 3 orthogonal directions to minimize the effects of diffusion anisotropy. The total acquisition time for DWI was approximately 4 to 6 minutes, depending on patient’s breathing frequency.

### Image analysis

The MADC program within the vendor-supplied FuncTool software was employed for the analysis of DWI images transferred to the GE Healthcare Advantage Workstation 4.6. The DWI images were analyzed by two of the authors without knowing the clinical or laboratory information by manually drawing regions of interest (ROI).

**Fig. 1:**
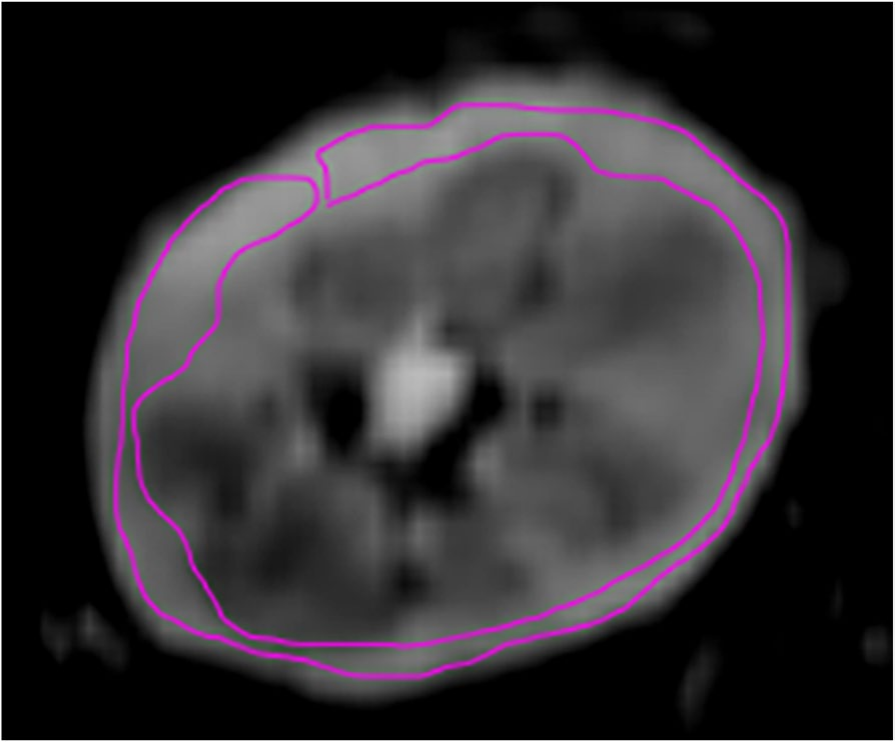
A typical example of region-of-interest delineation at a slice near the hilum in a kidney transplant on *b* = 0 image

As previously reported [5], a large ROI covering the entire allograft cortex was delineated on the six central slices close to the hilum in *b* = 0 images, resulting in six cortical ROIs for each allograft. A typical example of ROI delineation was presented in Fig. 1. A qualified radiologist (Y.M.Y., with 5 years of experience in abdominal MRI) manually drew the ROI, which was then confirmed by an experienced radiologist (L.J.Z., with over 20 years of experience in abdominal MRI). The cortical ROI readings were then averaged to obtain the corresponding DWI parameters for the allograft cortex.

The imaging signals were analyzed using both the monoexponential and biexponential models. Specifically, both *b* = 0 and the 10 nonzero *b*-values were fitted into the monoexponential model to obtain the total apparent diffusion coefficient (ADC_T_): S_b_/S_0_ = exp (-b ADC_T_), where *S*_b_ represents the signal intensity at a given *b*-value and *S*_0_ signifies the signal intensity at *b* = 0 s/mm^2^. The IVIM-derived parameters, including true diffusion (D), pseudo-diffusion (D*), and perfusion fraction (fp), were calculated using the following biexponential model: *S*_b_/*S*_0_ = (1-fp × exp (-b × D) + fp × exp (-b × [D + D*])). A segmented fit algorithm with constraints was applied, estimating the initial D value solely from *b*-values > 200 s/mm^2^. Subsequently, the resulting D was maintained as a fixed parameter to fit the missing values of D* and fp [6].

### Biochemical measurements, follow-up, and outcomes

Patient demographics and clinical and laboratory information at the time of DWI were collected from the electronic health records. The following data were recorded: age, sex, causes for end-stage kidney disease, serum creatinine, 24-h proteinuria, immunosuppression regimen, hemoglobin, albumin, and hematocrit. Allograft function was assessed with the estimated glomerular filtration rate (eGFR) using the creatinine-based Chronic Kidney Disease Epidemiology Collaboration equation [7]. Patients were regularly followed up every 3 to 6 months at the outpatient clinic post-discharge. The primary outcome was composite events including eGFR decline > 30% or the initiation of renal replacement therapy or re-transplantation, as previously reported [8]. For those who died, the last available eGFR was used to assess the primary outcome.

### Statistical analysis

Continuous variables were presented as mean ± standard deviation or median with interquartile range, as appropriate. Categorical variables were expressed as numbers and percentages. The optimal cut-off points for eGFR, proteinuria, and DWI parameters were determined based on the “maximally selected rank statistics” using the package prodlim for R (http://www.r-project.org/) as proposed previously [9]. This technique allows the distinction of a low- and high-risk group of patients by offering a cut-off point of the predictor while avoiding multiple testing in the meantime. Cumulative kidney allograft survival rate was estimated using the Kaplan-Meier method and compared using the log-rank test. Variables with a *p*-value < 0.05 in the univariate analysis were adopted into the multivariable Cox proportional-hazards regression model. To evaluate whether the model’s risk prediction could be improved by incorporating DWI parameters, we compared the area under the receiver-operating characteristic curve (AUROC) of the clinical model, the DWI model, and the composite model using DeLong’s test. Statistical significance was indicated by a two-sided *p*-value < 0.05.

## Results

### Characteristics of the study population

Patient demographics, clinical parameters, and laboratory findings are summarized in Table 1. A total of 97 patients were finally included, including 69 males and 28 females with a mean age of 38 years. The baseline median serum creatinine, eGFR, and proteinuria were 1.53 mg/dL, 56 mL/min/1.73 m^2^, and 0.39 g/24 h, respectively. The great majority (80.41%) of patients were on a triple immunosuppressive regimen consisting of prednisone, tacrolimus, and mycophenolic acid. Causes for end-stage kidney diseases were unknown in 82.47% patients. During a median follow-up time of 98 months (interquartile range, 44–103 months), a total of 45 patients achieved the primary outcome, including eGFR decline > 30% in 9 patients, return to dialysis in 34 patients, and re-transplantation in 2 patients.

**Table 1:** Demographics, clinical parameters, and laboratory findings of the cohort

Characteristics	Results
Age (years)^a^	38 ± 10
Sex (male) (*n*, %)	69 (71.13)
Baseline serum creatinine (mg/dL)^b^	1.53 (1.15–2.18)
Baseline eGFR (mL/min/1.73 m^2^)^b^	56 (35–77.5)
Proteinuria (g/24 h)^b^	0.39 (0.30–0.80)
Hemoglobin (g/dL)^a^	12.1 ± 2.5
Hematocrit^a^	0.369 ± 0.069
Albumin (g/L)^a^	43.3 ± 5.7
Immunosuppressive regimen	
Prednisone + mycophenolic acid + tacrolimus (*n*, %)	78 (80.41)
Prednisone + mycophenolic acid + cyclosporine A (*n*, %)	6 (6.19)
Others (*n*, %)	13 (13.40)
Causes for end-stage kidney disease	
Unknown (*n*, %)	80 (82.47)
Glomerulonephritides (*n*, %)	13 (13.40)
Other (*n*, %)	4 (4.13)
Follow-up time (months)^b^	98 (44–103)

^a^Indicated data are presented with mean ± standard deviation, whereas ^b^ denoted data expression as median with interquartile range

### Determination of optimal cut-off points for binary classifiers

**Fig. 2:**
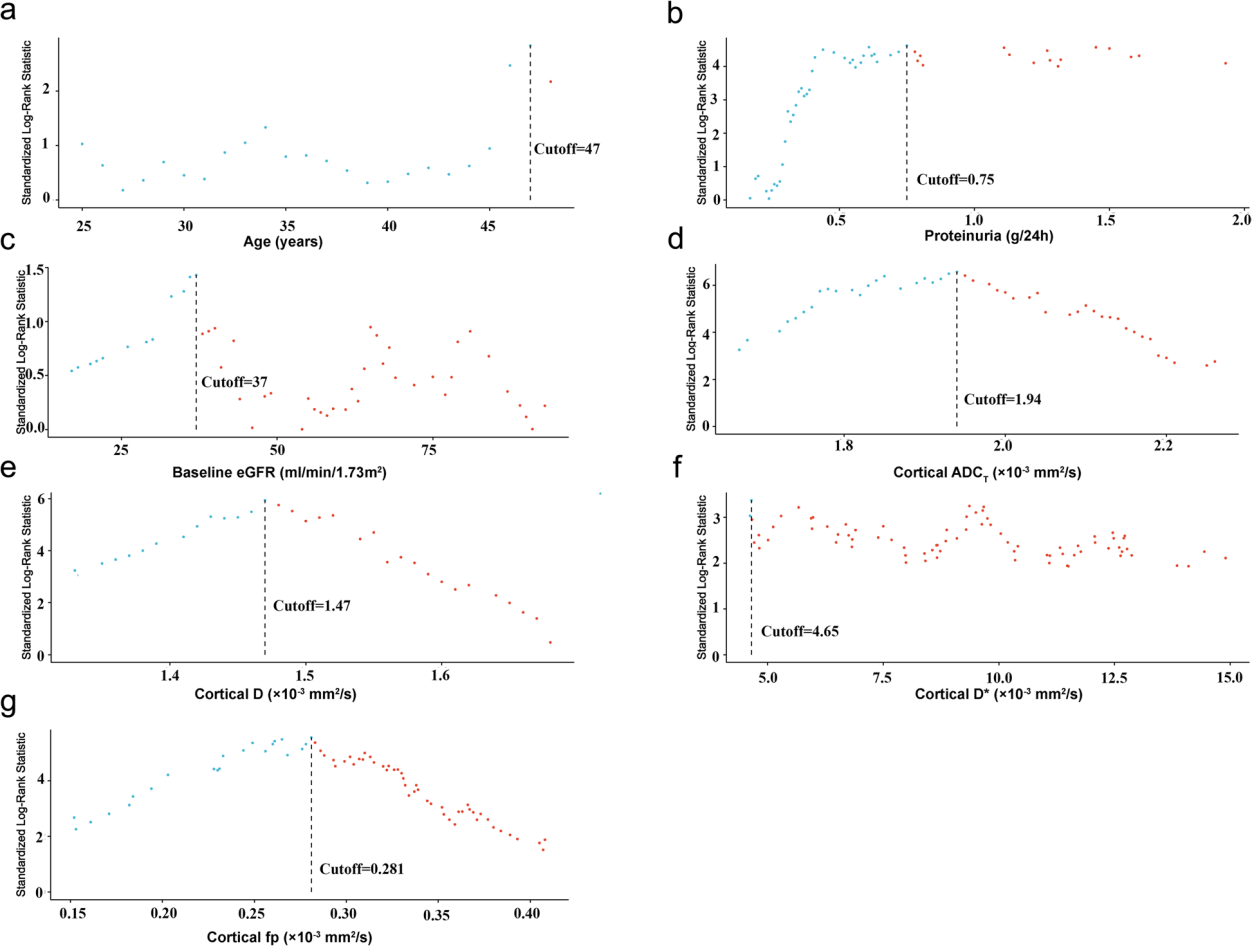
Determination of the optimal cut-off points for age (**a**), proteinuria (**b**), eGFR (**c**), cortical ADC_T_ (**d**), cortical D (**e**), cortical D* (**f**), and cortical fp (**g**) based on the “maximally selected rank statistics”

As shown in Fig. 2, the optimal cut-off points to assess the primary outcome as determined by the “maximally selected rank statistics” for ADC_T_, D, D*, and fp in the cortex were 1.94 × 10^−3^ mm^2^/s, 1.47 × 10^−3^ mm^2^/s, 4.65 × 10^−3^ mm^2^/s, and 0.281, respectively. Similarly, the optimal cut-off points for patient age, baseline eGFR, and proteinuria were 47 years, 37 mL/min/1.73 m^2^, and 0.75 g/24 h, respectively.

### Kaplan-Meier survival curves

**Fig. 3:**
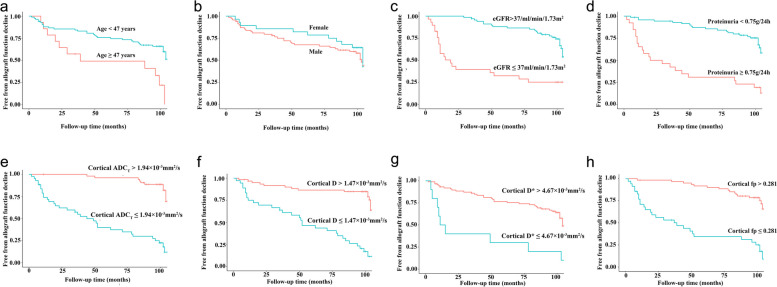
Kaplan-Meier curves of kidney allograft function decline stratified by patient age (**a**, HR = 2.48, *p* = 0.009), sex (**b**, HR = 1.32, *p* = 0.43), baseline estimated glomerular filtration rate (eGFR, **c**, HR = 4.94, *p* < 0.001), proteinuria (**d**, HR = 4.25, *p* < 0.001), cortical ADC_T_ (**e**, HR = 8.30, *p* < 0.001 ), cortical D (**f**, HR = 6.17, *p* < 0.001), cortical D* (**g**, HR = 4.29, *p* < 0.001), and cortical perfusion fraction (**h**, HR = 5.24, *p* < 0.001)

Patients were dichotomized into two groups based upon the cut-off points determined above. Kaplan-Meier curves (Fig. 3) showed that higher age (hazard ratio [HR] = 2.48, *p* = 0.009), lower baseline eGFR (HR = 4.94, *p* < 0.001), higher proteinuria (HR = 4.25, *p* < 0.001), lower cortical ADC_T_ (HR = 8.30, *p* < 0.001), lower cortical D (HR = 6.17, *p* < 0.001), lower cortical D* (HR = 4.29, *p* < 0.001), and lower cortical fp (HR = 5.24, *p* < 0.001) were all associated with the primary outcome. Nonetheless, kidney allograft survival for patients with different sex was similar (HR = 1.32, *p* = 0.43).

### Construction and comparisons of predictive models

We constructed a total of three predictive models using multivariable analysis. Specifically, the Model 1 (clinical model) was comprised of patient age, baseline eGFR, and proteinuria; the Model 2 (DWI model) included cortical ADC_T_, cortical D, cortical D*, and cortical fp; and the Model 3 (composite model) encompassed all the parameters in the Model 1 and Model 2. The results of each model for predicting the primary outcome are shown in Table 2. Multivariable analysis showed that the Model 3 included cortical D (HR = 3.93, *p* = 0.001) and cortical fp (HR = 2.85, *p* = 0.006), in addition to baseline eGFR (HR = 3.52, *p* = 0.002) and proteinuria (HR = 2.94, *p* = 0.003).

**Table 2:** Different models for predicting kidney allograft function decline by multivariable Cox regression analysis

Models	HR (95% *CI*)	*p*
Model 1		
Age, years (≥ 47 vs < 47)	2.21 (1.10–4.45)	0.03
Baseline eGFR, mL/min/1.73 m^2^ (≤ 37 vs > 37)	4.58 (2.44–8.62)	< 0.001
Proteinuria, g/24 h (≥ 0.75 vs < 0.75)	3.75 (2.02–6.93)	< 0.001
Model 2		
Cortical ADC_T_, ×10^−3^ mm^2^/s (≤ 1.94 vs > 1.94)	2.58 (1.03–6.48)	0.04
Cortical D, ×10^−3^ mm^2^/s (≤ 1.47 vs > 1.47)	3.00 (1.34–6.73)	0.008
Cortical D*, ×10^−3^ mm^2^/s (≤ 4.57 vs > 4.57)	3.96 (1.77–8.89)	0.001
Cortical fp (≤ 0.281 vs > 0.281)	3.48 (1.80–6.74)	< 0.001
Model 3		
Age, years (≥ 47 vs < 47)	1.35 (0.60–3.03)	0.48
Baseline eGFR, mL/min/1.73 m^2^ (≤ 37 vs > 37)	3.52 (1.61–7.71)	**0.002**
Proteinuria, g/24 h (≥ 0.75 vs < 0.75)	2.94 (1.43–6.08)	**0.003**
Cortical ADC_T_, ×10^−3^ mm^2^/s (≤ 1.94 vs > 1.94)	1.97 (0.76–5.09)	0.16
Cortical D, ×10^−3^ mm^2^/s (≤ 1.47 vs > 1.47)	3.93 (1.81–8.51)	**0.001**
Cortical D*, ×10^−3^ mm^2^/s (≤ 4.57 vs > 4.57)	2.08 (0.89–4.88)	0.09
Cortical fp (≤ 0.281 vs > 0.281)	2.85 (1.34–6.05)	**0.006**

*CI* confidence interval: *HR* hazard ratio

**Fig. 4:**
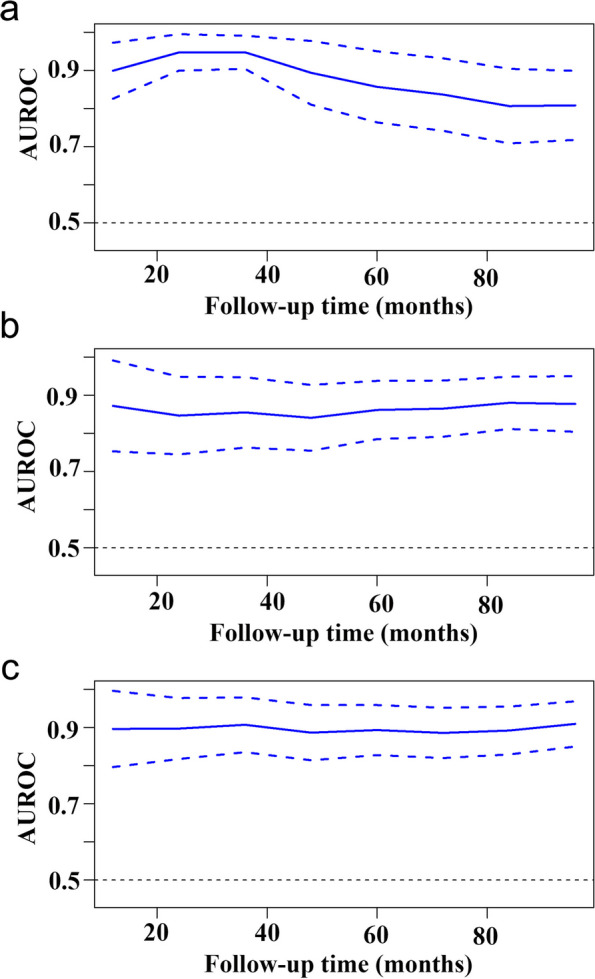
Time-dependent area under the receiver-operating characteristic curve (AUROC) for the clinical model (Model 1, **a**), DWI model (Model 2, **b**), and composite model (Model 3, **c**) during the follow-up time. The solid line represents the AUROC value, and the two dotted lines denote the corresponding 95% confidence intervals

**Fig. 5:**
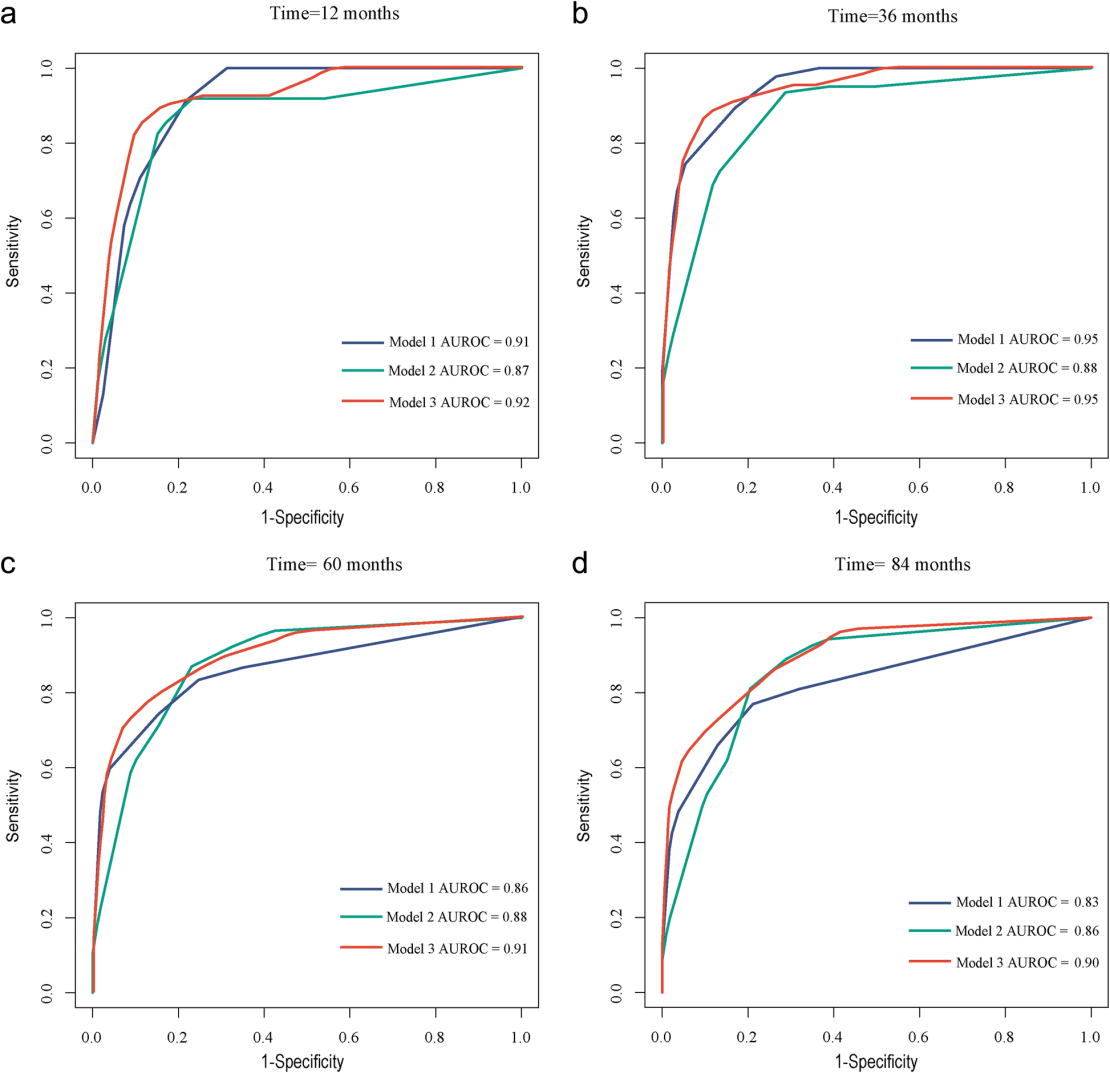
Receiver-operating characteristic curves for Model 1, Model 2, and Model 3 at 12-month (**a**), 36-month (**b**), 60-month (**c**), and 84-month (**d**) follow-up time points

The changes of AUROC with different follow-up time for each model are presented in Fig. 4. It can be observed that the AUROC for Model 1 gradually decreased with the follow-up time > 40 months, whereas the Model 2 and Model 3 maintained relatively stable AUROC. We then set the follow-up time to 12 months, 36 months, 60 months, and 84 months to compare the predictive ability of each model by calculating the AUROC. As presented in Fig. 5, the AUROCs of Model 1 and Model 2 were not statistically significant at 12-month (0.91 vs 0.87, *p* = 0.83), 36-month (0.95 vs 0.88, *p* = 0.53), 60-month (0.86 vs 0.88, *p* = 0.83), and 84-month follow-up (0.83 vs 0.86, *p* = 0.34). In comparison, the AUROCs of Model 3 were significantly higher than those of Model 1 at the follow-up time of 60 months (0.91 vs 0.86, *p* = 0.02) and 84 months (0.90 vs 0.83, *p* = 0.007). The AUROCs of Model 3 were comparable with those of Model 1 at 12-month (0.92 vs 0.91, *p* = 0.07) and 36-month (0.95 vs 0.95, *p* = 0.13) follow-up.

## Discussion

We evaluated the added value of cortical DWI parameters for predicting allograft function decline in a cohort of 97 patients with a median follow-up of 98 months. The results showed that cortical D and fp were predictors of allograft function decline independent of baseline eGFR and proteinuria. Furthermore, the addition of these two IVIM-DWI parameters to a clinical model consisting of baseline eGFR and proteinuria may provide incremental prognostic value for allograft function decline with long-term (≥ 60 months) follow-up.

The finding that baseline eGFR and proteinuria are predictors of allograft function decline is not surprising, given that abundant prior investigations have consistently demonstrated that higher proteinuria and declining kidney function as indicated by lower eGFR are strong predictors of unfavorable outcome for both native kidneys and kidney transplants [10, 11]. Therefore, measuring serum creatinine and proteinuria is a cost-effective and ubiquitous clinical approach that assist in the clinical management and prognostication for patients with kidney transplants.

A novel finding of the present study is that cortical D and fp, both of which are parameters obtained through IVIM analysis of the DWI signals, are independent risk factors associated with the decline of allograft function. We are aware that several earlier studies have explored the prognostic significance of ADC_T_ for kidney outcome with conflicting results. For instance, both the study by Sugiyama et al. [12] in a cohort of 91 patients with chronic kidney disease and the study by Berchtold et al. [13] in a mixed cohort of 197 patients with both chronic kidney disease and kidney allograft dysfunction suggested that cortical ADC_T_ did not predict the decline of kidney function. Nonetheless, Srivastava’s group [14] demonstrated that baseline cortical ADC_T_ was associated with change in eGFR over time. Interestingly, the associations between ADC_T_ and allograft function decline identified in Kaplan-Meier curves disappeared in multivariable analysis in the present study. We attributed this to the notion that ADC_T_ encompasses both D and D* caused by microcapillary perfusion. The work [15] of Cheng et al. uncovered that D outperformed ADC_T_ in assessing underlying kidney pathologic changes, suggesting ADC_T_ might be less sensitive than D for evaluating kidney microstructural alterations.

To the best of our knowledge, the significance of IVIM-DWI parameters for predicting kidney outcome has not been previously explored. An increasing number of studies have suggested that both the D and fp were correlates of kidney pathologic changes, particularly kidney interstitial fibrosis [16]. The cortical D measures water molecule movement that is predominantly reflective of the extent of interstitial fibrosis. The fp has been shown to be a robust and reproducible measure for assessing tissue perfusion status [17]. Earlier studies have indicated a significant correlation between cortical fp and allograft perfusion, as well as serving as a reflection of kidney microvessel density [18]. In congruence with our findings, prior investigations demonstrated that cortical D and fp were intimately correlated with kidney interstitial fibrosis [19], which is considered a reliable histologic indicator for kidney function deterioration. The D* has been noted to suffer from low reproducibility as indicated by suboptimal inter-reader agreement, so some studies simply did not report D* results [20].

We observed that DWI parameters had comparable prognostic capability to model 1, and they did not appear to add prognostic significance in those with short-term follow-up. The usefulness of DWI parameters for predicting kidney allograft function deterioration is mostly confined to those with long-term follow-up. We interpret this finding as suggesting that the clinical utility of multi-b DWI for short-term prognostication is limited. However, it does offer additional benefits for managing patients with kidney transplants in the long term. In line with this, a previous study [21] reported that DWI can help reduce unnecessary allograft biopsies by improving the detection rate of allografts with underlying pathologic alterations. Thus, multi-b DWI could potentially be incorporated into the armamentarium for transplant surgeons and radiologists caring for patients with a kidney transplant with long-term follow-up.

Although we observed a relatively large cohort for an extended follow-up time, the limitations of this study must be acknowledged. First, the retrospective nature of this study may introduce potential selection biases that may confound the study results. Consequently, extrapolation of the study results to the kidney transplant population with different clinical characteristics from our cohort may be difficult. For example, the causes of end-stage kidney disease in the majority of patients in this cohort were unknown, and it remains unclear whether this would affect the DWI’s prognostic value. In addition, the added value of DWI for kidney transplants with long-term follow-up should be further investigated in the future through cost-effectiveness and comparative analysis with ultrasound, which is currently the most widely used imaging modality for the evaluation of renal allografts.

## Conclusion

In conclusion, we have demonstrated that both cortical D and cortical fp were predictive of allograft outcome, independent of baseline eGFR and proteinuria. Additionally, incorporating cortical D and fp into a clinical model with baseline eGFR and proteinuria may add prognostic value for long-term allograft function decline. The cost-effectiveness of integrating multi-b DWI to predict kidney function decline should be validated in the future prior to its clinical application.

## Data Availability

The datasets used for analyses during the current study are available from the corresponding author on reasonable request.

## Abbreviations

AUROC: Area under the receiver-operating characteristic curve
DWI: Diffusion-weighted imaging
eGFR: Estimated glomerular filtration rate
HR: Hazard ratio
IVIM: Intravoxel incoherent motion
ROI: Region of interest
